# Supplementary material for: Matrix condition mediates the effects of habitat fragmentation on species extinction risk
Source: Nat Commun. 2022 Feb 1;13:595. doi: 10.1038/s41467-022-28270-3 (PMC8807630; doi:10.1038/s41467-022-28270-3)
Supplement: Supplementary file 1 — Supplementary information [file 41467_2022_28270_MOESM1_ESM.pdf]

Supplementary information for

Ramírez-Delgado et al.

Matrix condition mediates the effects of habitat fragmentation on species extinction risk

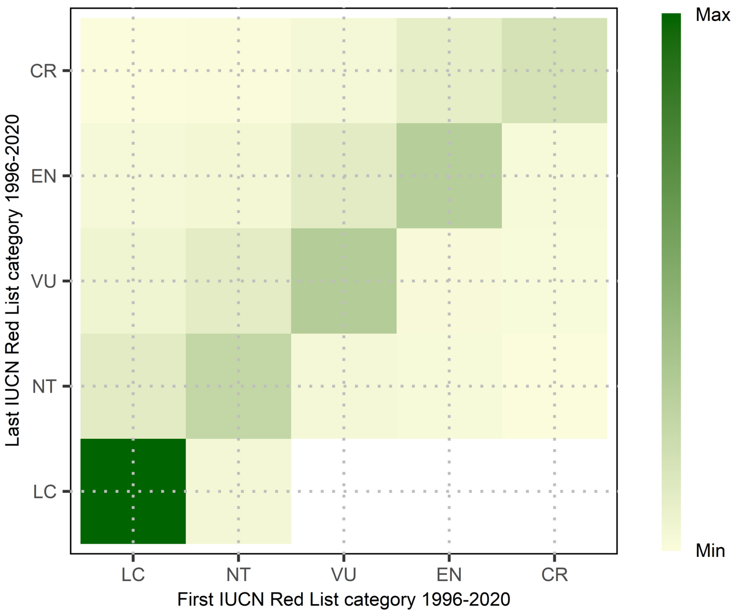

Supplementary Fig. 1. Transition matrix of extinction risk categories for terrestrial mammals between 1996 and 2020. The plot shows the transition matrix of the first and last Red List category reported between 1996 and 2020. The colour scheme represents the relative frequency of individual species in each transition. Acronyms refer to the IUCN Red List categories, including Least Concern (LC), Near Threatened (NT), Vulnerable (VU), Endangered (EN), and Critically Endangered (CR). The relative frequency was arcsine square-root-transformed for visual purposes and to avoid variance instability when handling proportions close to zero. Source data are provided as a Source Data file.

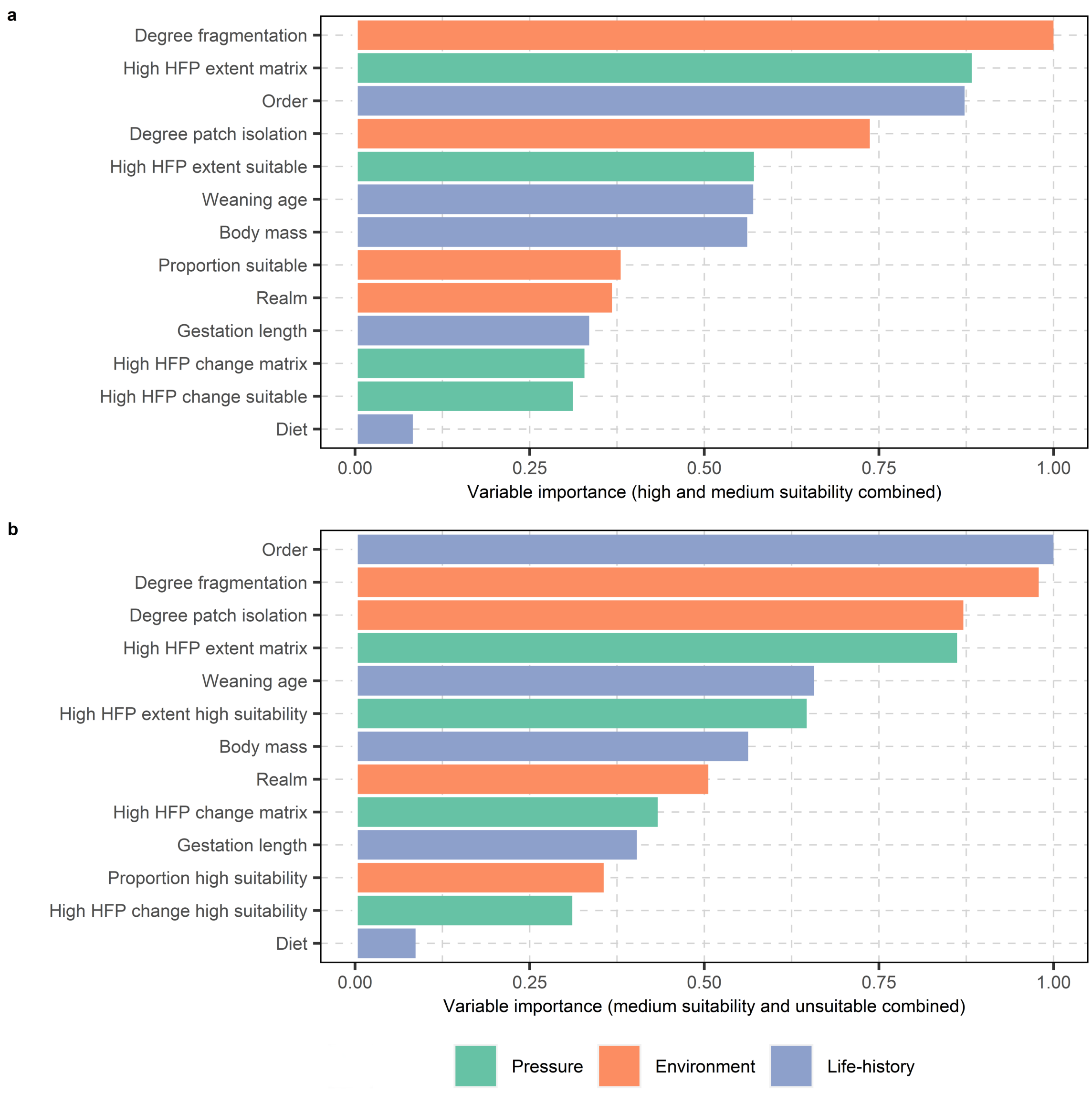

Supplementary Fig. 2. Sensitivity analysis of selected variables for the prediction of extinction risk transitions in terrestrial mammals. a Relative importance of each predictor when the extent of suitable habitat is represented by high and medium habitat suitability combined, and the extent of the matrix by unsuitable habitat alone. **b** Relative importance of each predictor when the extent of suitable habitat is represented by high habitat suitability, and the extent of the matrix by medium habitat suitability and unsuitable habitat combined. Variables are colour-coded according to their broad class (human pressure, environment, and life-history). The description of each variable is given in the Table 1 of the main manuscript. High levels of the human footprint (HFP) included values of 3 or above. Source data are provided as a Source Data file.

Supplementary Table 1. Cross-validation results of the Random Forest models for the prediction of extinction risk transitions in terrestrial mammals. *Cross-validation results when the extent of suitable habitat is represented by high and medium habitat suitability combined, and the extent of the matrix by unsuitable habitat alone. †Cross-validation results when the extent of suitable habitat is represented by high habitat suitability, and the extent of the matrix by medium habitat suitability and unsuitable habitat combined. The cross-validation was performed in terms of proportion of correctly classified species (accuracy), proportion of correctly classified high-risk species (sensitivity), proportion of correctly classified low-risk species (specificity), and the true skill statistic (TSS = sensitivity + specificity – 1). Source data are provided as a Source Data file.

Model	Accuracy (%)	Sensitivity (%)	Specificity (%)	TSS
Extinction risk transitions ~ High and medium suitability combined*	81.2	60.5	90.9	0.51
Extinction risk transitions ~ Medium suitability and unsuitable combined†	82.0	61.1	91.7	0.53

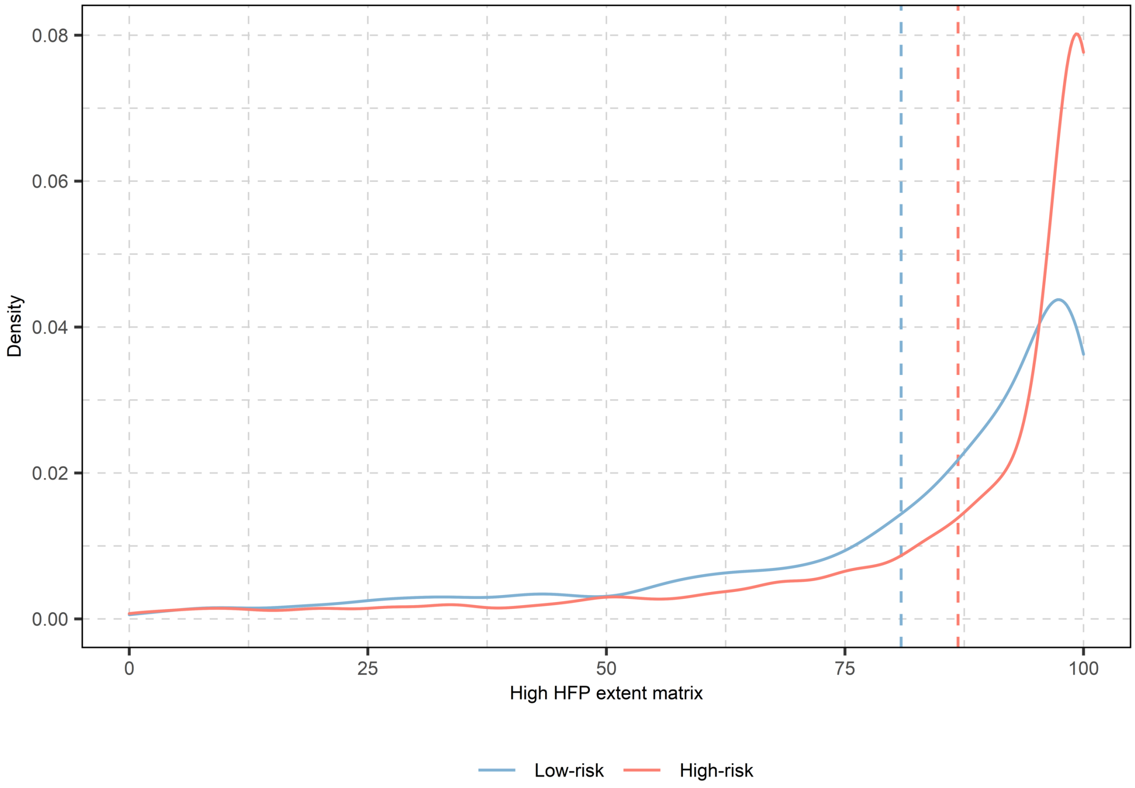

Supplementary Fig. 3. Distribution of the matrix condition in low-risk and high-risk species at a global scale. The plot represents the frequency distribution of the extent of high human footprint values within the matrix of 4,329 species (68% low-risk species and 32% high-risk species). Species with ranges that did not overlap with the human footprint (2% of 4,426 terrestrial mammals in our sample) were excluded from the calculations. Blue and red lines refer to low-risk and high-risk species, as reported in legend. Dashed lines denote mean of distributions. High levels of the human footprint (HFP) included values of 3 or above. Source data are provided as a Source Data file.

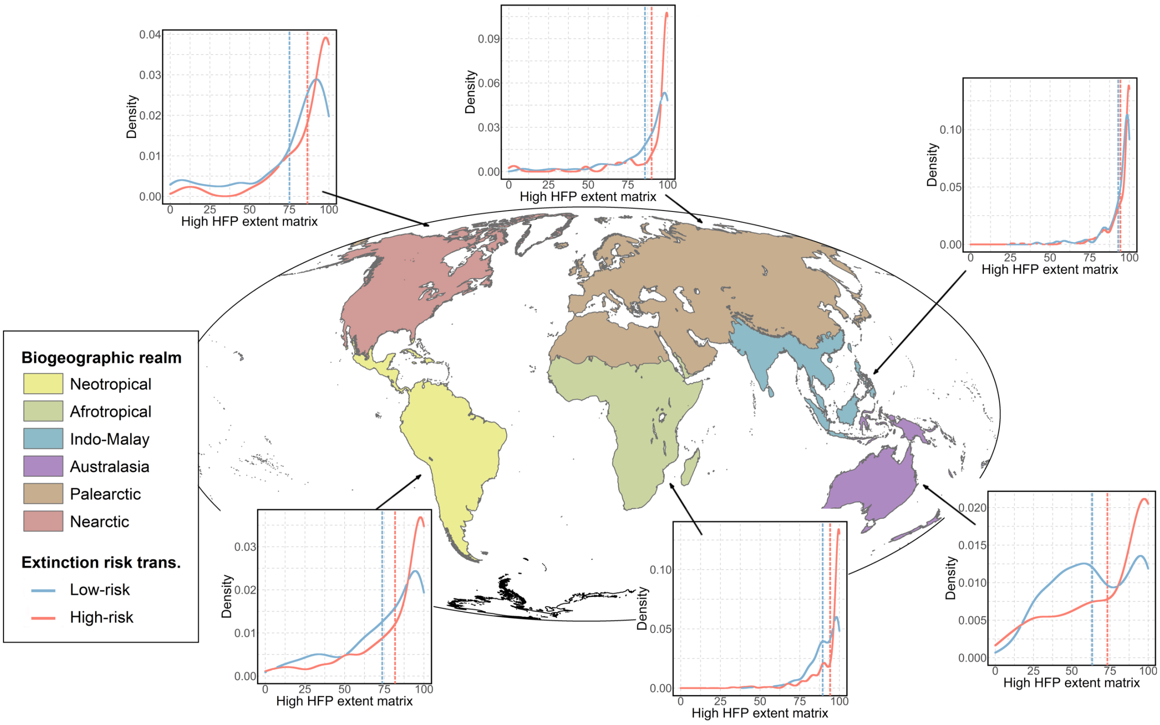

Supplementary Fig. 4. Distribution of the matrix condition in low-risk and high-risk species at the scale of individual biogeographic realms. The plots represent the frequency distribution of the extent of high human footprint values within the matrix of 1.109 species in the Neotropical realm (68% low-risk species and 32% high-risk species), 990 species in the Afrotropical realm (65% low-risk species and 35% high-risk species), 596 species in the Indo-Malay realm (52% low-risk species and 48% high-risk species), 552 species in the Australasia realm (58% low-risk species and 42% high-risk species), 498 species in the Palearctic realm (79% low-risk species and 21% high-risk species), and 286 species in the Nearctic realm (83% low-risk species and 17% high-risk species). The Oceania realm included a total of 4 species only (17% low-risk species and 83% high-risk species), which are not depicted on the map. Species with ranges that overlapped with two or more realms (9% of 4,426 terrestrial mammals in our sample) were excluded from the calculations. Blue and red lines refer to low-risk and high-risk species, as reported in legend. Dashed lines denote mean of distributions. High levels of the human footprint (HFP) included values of 3 or above. Source data are provided as a Source Data file.

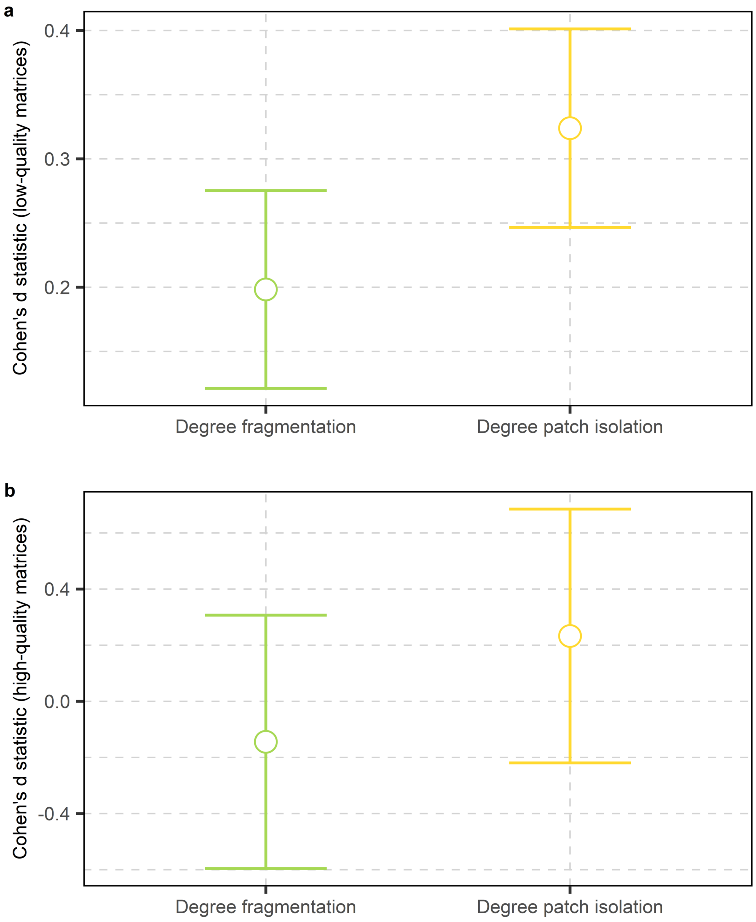

Supplementary Fig. 5. Effect size (Cohen's d statistic) of the degree of habitat fragmentation and the degree of patch isolation between low-risk and high-risk species with a low-quality matrix and a high-quality matrix. a Effect size of the degree of fragmentation and the degree of patch isolation between low-risk and high-risk species with a matrix of low-quality habitat. **b** Effect size of the degree of fragmentation and the degree of patch isolation between low-risk and high-risk species with a matrix of high-quality habitat. Green and yellow points represent the effect sizes or the standardized difference of mean values (Cohen's d statistic) of the degree of fragmentation and the degree of patch isolation between low-risk and high-risk species, respectively. Error bars represent 95% confidence intervals for each effect size calculated. Low-quality matrices included species with proportions > 84.2% of the extent of their matrix overlapping with high human footprint values (*n* = 1,815 low-risk species and 1,027 high-risk species). High-quality matrices included species with proportions < 15.8% of the extent of their matrix overlapping with high human footprint values (*n* = 60 low-risk species and 29 high-risk species). High levels of the human footprint included values of 3 or above. Source data are provided as a Source Data file.
