# Supplementary material for: Matrix condition mediates the effects of habitat fragmentation on species extinction risk
Source: Nat Commun. 2022 Feb 1;13:595. doi: 10.1038/s41467-022-28270-3 (PMC8807630; doi:10.1038/s41467-022-28270-3)
Supplement: Supplementary file 3 — Reporting Summary [file 41467_2022_28270_MOESM3_ESM.pdf]

nature portfolio

Corresponding author(s): Juan Pablo Ramírez-Delgado

Last updated by author(s): Dec 27, 2021

Reporting Summary

Nature Portfolio wishes to improve the reproducibility of the work that we publish. This form provides structure for consistency and transparency in reporting. For further information on Nature Portfolio policies, see our Editorial Policies and the Editorial Policy Checklist.

Statistics

For all statistical analyses, confirm that the following items are present in the figure legend, table legend, main text, or Methods section.

n/a	Confirmed	
☐	☒	The exact sample size (*n*) for each experimental group/condition, given as a discrete number and unit of measurement
☐	☒	A statement on whether measurements were taken from distinct samples or whether the same sample was measured repeatedly
☐	☒	The statistical test(s) used AND whether they are one- or two-sided<br>*Only common tests should be described solely by name; describe more complex techniques in the Methods section.*
☐	☒	A description of all covariates tested
☐	☒	A description of any assumptions or corrections, such as tests of normality and adjustment for multiple comparisons
☐	☒	A full description of the statistical parameters including central tendency (e.g. means) or other basic estimates (e.g. regression coefficient) AND variation (e.g. standard deviation) or associated estimates of uncertainty (e.g. confidence intervals)
☐	☒	For null hypothesis testing, the test statistic (e.g. *F*, *t*, *r*) with confidence intervals, effect sizes, degrees of freedom and *P* value noted<br>*Give P values as exact values whenever suitable.*
☒	☐	For Bayesian analysis, information on the choice of priors and Markov chain Monte Carlo settings
☒	☐	For hierarchical and complex designs, identification of the appropriate level for tests and full reporting of outcomes
☐	☒	Estimates of effect sizes (e.g. Cohen's *d*, Pearson's *r*), indicating how they were calculated

Our web collection on statistics for biologists contains articles on many of the points above.

Software and code

Policy information about availability of computer code

Data collection	Data on Red List categories over time were collected in R v4.1.0, using the package 'rredlist' v0.7.0. The code used for collecting data on Red List categories is available at https://github.com/juanxramirez/Matrix_condition and at https://doi.org/10.5281/zenodo.5803377. The other datasets that support the findings of this study were collected from published sources, cited in the Methods section and listed in Table 1. No software was used to collect these data.
Data analysis	All spatial analyses were performed in python v3.7.10, using the ArcPy processing module from ArcGIS Pro v2.8.2.<br>Statistical analyses were performed in R v4.1.0, using the packages 'randomforest' v4.6.14, 'caret' v6.0.88, 'iml' v0.10.1, and 'effsize' v0.8.1.<br>All the code used in this work has been deposited in GitHub at https://github.com/juanxramirez/Matrix_condition, and mirrored in Zenodo at https://doi.org/10.5281/zenodo.5803377.

For manuscripts utilizing custom algorithms or software that are central to the research but not yet described in published literature, software must be made available to editors and reviewers. We strongly encourage code deposition in a community repository (e.g. GitHub). See the Nature Portfolio guidelines for submitting code & software for further information.

Data

Policy information about availability of data

All manuscripts must include a data availability statement. This statement should provide the following information, where applicable:

- Accession codes, unique identifiers, or web links for publicly available datasets
- A description of any restrictions on data availability
- For clinical datasets or third party data, please ensure that the statement adheres to our policy

The input dataset used to run our models of extinction risk and that support the findings of this study has been deposited in GitHub (https://github.com/

juanxramirez/Matrix_condition), and mirrored on Zenodo (https://doi.org/10.5281/zenodo.5803377). The Human Footprint dataset used in this study is available for download at https://doi.org/10.5061/dryad.3tx95x6d9. Habitat suitability models for the world's terrestrial mammals are available upon request from the model developers at https://globalmammal.org/habitat-suitability-models-for-terrestrial-mammals/. Data on Red List categories through time are available upon request at https://apiv3.iucnredlist.org/. In this study, these data were accessed from R99, using the package 'rredlist'104. Data on genuine changes in the Red List categories are available in Hoffmann et al.53, and at https://www.iucnredlist.org/resources/summary-statistics. The other datasets that support the findings of this study derive from published sources, cited in the Methods section and listed in Table 1.

Field-specific reporting

Please select the one below that is the best fit for your research. If you are not sure, read the appropriate sections before making your selection.

- [ ] Life sciences
- [ ] Behavioural & social sciences
- [x] Ecological, evolutionary & environmental sciences

For a reference copy of the document with all sections, see nature.com/documents/nr-reporting-summary-flat.pdf

Ecological, evolutionary & environmental sciences study design

All studies must disclose on these points even when the disclosure is negative.

Study description	We measured the influence of the condition of the matrix, represented by the extent of high human footprint levels surrounding patches of suitable habitat, on the effects of habitat fragmentation for determining transitions of extinction risk in terrestrial mammals.
Research sample	We used habitat suitability models developed by Rondinini et al. 2011 (https://doi.org/10.1098/rstb.2011.0113) to represent the extent of suitable habitat patches and the extent of the matrix of the world's terrestrial mammals. These models were built for the year 2000 at a spatial resolution of 300 m. Spatially explicit data on the condition of the matrix, as represented by the extent of high human footprint levels within the matrix, was obtained from the recently updated global human footprint maps developed by Williams et al. 2020 (https://doi.org/10.1016/j.oneear.2020.08.009). These maps represent the most comprehensive global distribution of changing human pressure on the environment at 1 km resolution between 2000 and 2013. We represented changes in the risk of extinction of terrestrial mammals between 1996 and 2020 using the information available from the IUCN Red List of Threatened Species (https://apiv3.iucnredlist.org/), the retrospective Red List Assessments published in Hoffmann et al. 2010 (https://doi.org/10.1126/science.1194442), and the IUCN list of genuine changes in the conservation status of mammals species (https://www.iucnredlist.org/resources/summary-statistics).
Sampling strategy	Sample sizes were chosen based on the number of species with a defined level of habitat suitability and a defined transition of extinction risk during the study period (i.e. between 1996 and 2020).
Data collection	The Human Footprint dataset used in this study is available for download at https://doi.org/10.5061/dryad.3tx95x6d9. Habitat suitability models for the world's terrestrial mammals are available upon request from the model developers at https://globalmammal.org/habitat-suitability-models-for-terrestrial-mammals/. Data on Red List categories through time are available upon request at https://apiv3.iucnredlist.org/. In this study, these data were accessed from R, using the package 'rredlist'. Data on genuine changes in the Red List categories are available in Hoffmann et al. 2010 (https://doi.org/10.1126/science.1194442), and at https://www.iucnredlist.org/resources/summary-statistics. The other datasets that support the findings of this study derive from published sources, cited in the Methods section and listed in Table 1. J.P.R.-D. collected all the data.
Timing and spatial scale	The reference time period for our analysis is 1996-2020. This time frame was used because data on Red List categories are consistent over time and currently available for this time period (see https://apiv3.iucnredlist.org/, https://doi.org/10.1126/science.1194442, and https://www.iucnredlist.org/resources/summary-statistics). Spatially explicit data on the extent of suitable habitat and the extent of the matrix were built for the year 2000 at a spatial resolution of 300 m (see https://doi.org/10.1098/rstb.2011.0113). Spatially explicit data on the condition of the matrix was represented by the extent of high human footprint levels (human footprint values ≥ 3 out of 50) within the matrix for the years 2000 and 2013 at a spatial resolution of 1 km (see https://doi.org/10.1016/j.oneear.2020.08.009). The used geographic projection is World Mollweide.
Data exclusions	We excluded species without a defined level of habitat suitability, those not evaluated in the Red List, and those categorized as Data Deficient, Extinct and Extinct in the Wild in the last Red List assessment reported during the study period, as long as they have not shown a defined transition of extinction risk (see Fig. 1) along the study period.
Reproducibility	Our findings can be reproduce using the datasets and codes available at https://github.com/juanxramirez/Matrix_condition. The dataset and codes have also been made available at https://doi.org/10.5281/zenodo.5803377.
Randomization	We classified species into two groups of extinction risk, low-risk transitions and high-risk transitions, based on the first and last Red List category registered between 1996 and 2020. Low-risk transitions included species that retained a category of least concern, together with those species that moved from any higher category of threat to a lower category between 1996 and 2020. High-risk transitions included all species that retained a category of threatened or near threatened, together with those species that moved from any lower category of threat to a higher category between 1996 and 2020. We also defined two levels of quality of the matrix (low-quality matrices and high-quality matrices) based on the positive and negative effect that the matrix condition had on the probability of low-risk and high-risk transitions. Low-quality matrices were therefore represented by species with extents > 84.2% of their matrix overlapping with high human footprint values, while high-quality matrices by those species with extents < 15.8% of their matrix overlapping with high human footprint values. For our analyses, we used Random Forest models, which build multiple decision trees and combine the results to get a more accurate prediction. We optimized the number of trees to grow and the number of predictors sampled for splitting at each node from 3 repeats of 10-fold cross-validation, using 75% of the data as training data and 25% as test data in each model.

Blinding	Blinding was not relevant as we collected data from the literature.

Did the study involve field work? ☐ Yes ☒ No

Reporting for specific materials, systems and methods

We require information from authors about some types of materials, experimental systems and methods used in many studies. Here, indicate whether each material, system or method listed is relevant to your study. If you are not sure if a list item applies to your research, read the appropriate section before selecting a response.

Materials & experimental systems

n/a	Involved in the study	
☒	☐	Antibodies
☒	☐	Eukaryotic cell lines
☒	☐	Palaeontology and archaeology
☒	☐	Animals and other organisms
☒	☐	Human research participants
☒	☐	Clinical data
☒	☐	Dual use research of concern

Methods

n/a	Involved in the study	
☒	☐	ChIP-seq
☒	☐	Flow cytometry
☒	☐	MRI-based neuroimaging
